# Expression of Novel Alzheimer’s Disease Risk Genes in Control and Alzheimer’s Disease Brains

**DOI:** 10.1371/journal.pone.0050976

**Published:** 2012-11-30

**Authors:** Celeste M. Karch, Amanda T. Jeng, Petra Nowotny, Janet Cady, Carlos Cruchaga, Alison M. Goate

**Affiliations:** Department of Psychiatry and Hope Center for Neurological Disorders, Washington University School of Medicine, St Louis, Missouri, United States of America; Nathan Kline Institute and New York University School of Medicine, United States of America

## Abstract

Late onset Alzheimer’s disease (LOAD) etiology is influenced by complex interactions between genetic and environmental risk factors. Large-scale genome wide association studies (GWAS) for LOAD have identified 10 novel risk genes: *ABCA7*, *BIN1*, *CD2AP*, *CD33*, *CLU*, *CR1*, *EPHA1*, *MS4A6A*, *MS4A6E*, and *PICALM*. We sought to measure the influence of GWAS single nucleotide polymorphisms (SNPs) and gene expression levels on clinical and pathological measures of AD in brain tissue from the parietal lobe of AD cases and age-matched, cognitively normal controls. We found that *ABCA7, CD33,* and *CR1* expression levels were associated with clinical dementia rating (CDR), with higher expression being associated with more advanced cognitive decline. *BIN1* expression levels were associated with disease progression, where higher expression was associated with a delayed age at onset. *CD33, CLU,* and *CR1* expression levels were associated with disease status, where elevated expression levels were associated with AD. Additionally, *MS4A6A* expression levels were associated with Braak tangle and Braak plaque scores, with elevated expression levels being associated with more advanced brain pathology. We failed to detect an association between GWAS SNPs and gene expression levels in our brain series. The minor allele of rs3764650 in *ABCA7* is associated with age at onset and disease duration, and the minor allele of rs670139 in *MS4A6E* was associated with Braak tangle and Braak plaque score. These findings suggest that expression of some GWAS genes, namely *ABCA7*, *BIN1*, *CD33*, *CLU*, *CR1* and the *MS4A* family, are altered in AD brains.

## Introduction

Late onset Alzheimer’s disease (LOAD) is the most common form of dementia. AD is pathologically defined by extensive neuronal loss and the accumulation of extracellular amyloid plaques and intracellular neurofibrillary tangles in the brain. While the familial form of AD is associated with heritable mutations in the *APP*, *PSEN1*, and *PSEN2* genes, LOAD onset and progression appears to be influenced by complex interactions between genetic and environmental risk factors. Apolipoprotein ε4 (*APOE4*) is the strongest genetic risk factor for LOAD [1]–[4] but only accounts for 10–20% of LOAD risk suggesting that susceptibility to LOAD involves additional genetic and environmental risk factors.

In recent efforts to identify additional genetic risk factors for LOAD, large-scale genome-wide association studies (GWAS) have identified single nucleotide polymorphisms (SNP) in 10 genes: *ABCA7*, *BIN1*, *CD2AP*, *CD33*, *CLU*, *CR1*, *EPHA1*, *MS4A6A*, *MS4A4E*, and *PICALM* [5]–[10]. These genes fall into several functional pathways that are affected in AD: immune response (*CLU*, *CR1*, *ABCA7*, *MS4A* family, *CD33*, and *EPHA1*), cholesterol metabolism (*CLU* and *ABCA7*), and synaptic function (*PICALM*, *BIN1*, *CD33*, *CD2AP*, and *EPHA1*).

Despite the identification of numerous SNPs that occur in genes that function in pathways relevant to AD, we still know little of the specific functional impact of the LOAD GWAS SNPs and the specific role of these genes in AD. Thus, we sought to measure the influence of GWAS SNPs on gene expression in a cohort of AD cases and age-matched, cognitively normal control brains. We found that *ABCA7*, *BIN1*, *CD33*, *CLU*, *CR1*, and *MS4A6A* expression are associated with clinical and neuropathological measures of AD. The GWAS SNPs, however, were not associated with gene expression. Thus, we found that the expression patterns of some GWAS genes are altered in AD brains.

## Materials and Methods

### Subjects

Parietal lobes from European American, autopsy-confirmed AD (N = 73) and age-matched, cognitively normal control (N = 39) brains were obtained from the Charles F. and Joanne Knight Alzheimer’s Disease Research Center (Table 1). AD pathology was measured using Braak and Braak staging [11], [12]. Clinical dementia rating (CDR) is a clinical measure of dementia, which incorporates six domains of cognitive and functional abilities: memory, orientation, problem solving, community involvement, home, and personal care [13].

**Table 1:** Summary of brain samples.

Sample	N	Age (yrs)*	Male (%)	ApoE4+ (%)	Braak Tangle Score*	Braak Amyloid Score*
Case	73	87±7	42	41	4.6±1.6	2.7±0.8
Control	39	86±9	44	23	2.6±1.3	1.6±1.2

* Mean ± SD.

The Washington University IRB reviewed the Knight ADRC Neuropathology Core (from whom the brains were obtained) operating protocol as well as this specific study and determined it was exempt from approval. In the state of Missouri, individuals can give prospective consent for autopsy. Our participants provide this consent by signing the hospital's autopsy form. If the participant does not provide future consent before death the DPOA or next of kin provide it after death. All data were analyzed anonymously.

### RNA Extraction

RNA was extracted from brain tissue with an RNeasy kit (Qiagen) according to the manufacture’s protocol. Extracted RNA (10ug) was converted to cDNA by PCR using the High-Capacity cDNA Reverse Transcriptase kit (ABI). RNA integrity (RIN) was measured in an Agilent Bioanalyzer with an Agilent RNA Pico Kit (Table S1).

### Real Time Reactions

Gene expression was analyzed by real-time PCR using an ABI-7900 real-time PCR system. Taqman real-time PCR assays were utilized to quantify expression for the following genes: *ABCA7* (ABI: Hs01105094_m1), *AIF1* (ABI: Hs00610419_g1), *BIN1* (ABI: Hs00184913_m1), *BIN1*n (ABI : Hs01120896_m1), *CD2AP* (ABI: Hs00961451_m1), *CD33* (ABI: Hs00233544_m1), *CLU* ^1^ (ABI: Hs00156548_m1), *CLU* ^2^ (ABI: Hs00971653_m1), *CR1* (ABI: Hs00559342_m1), *EPHA1* (ABI: Hs00178313_m1), *GAPDH* (ABI: Hs02758991_g1 (VIC) and Hs99999905_m1 (FAM)), *GFAP* (ABI: Hs00909233_m1), *MAP2* (ABI: Hs00258900_m1), MS4A6A (ABI: Hs00223521_m1), *PICALM* (ABI: Hs00200318_m1). *BIN1*n is a probe specific to a neuronal isoform of *BIN1*. *CLU* ^1^ is a probe specific to exons 3–4 in *CLU*, and *CLU* ^2^ is a probe specific to exons 4–5 in *CLU*. Samples were run in triplicate with replicate samples analyzed in each plate to control for plate-to-plate variability. To avoid amplification interference, expression assays were run in separate wells from the housekeeping gene *GAPDH*.

Real-time data were analyzed by the comparative C_T_ method [14]. Average C_T_ values for each sample were normalized to the average C_T_ values for the housekeeping gene *GAPDH* (Figure S1). The resulting value was then corrected for assay efficiency. Samples with a standard error of 20% or less were subsequently analyzed. *GAPDH* expression was highly correlated with *PPIA* expression, an additional endogenous housekeeping gene (Figure S2); thus, all subsequent analyses used GAPDH expression as a control.

### Genotyping

Genomic DNA was extracted from the parietal lobe of AD and cognitively normal control brains using the DNeasy Blood and Tissue kit (Qiagen). SNPs were genotyped using Kaspar and Taqman genotyping assays. Kaspar assays were used for the following SNPs: rs11767557 (*EPHA1*), rs59335482 (*BIN1*) rs9349407 (*CD2AP*), rs38654444 (*CD33*), rs670173 (*CR1*). Taqman assays were used for the following SNPs: rs3764650 (*ABCA7*), rs744373 (*BIN1*), rs7982 (*CLU*), rs3818361 (*CR1*), rs610932 (*MS4A6A*), rs670139 (*MS4A4E*), rs3851179 (*PICALM*). SNPs were analyzed with a call rate of 95% or higher.

### Statistical Analysis

Relative gene expression values were log transformed to achieve a normal distribution (Figure S3). To identify covariates that influence the expression of each gene, a stepwise discriminant analysis was performed using CDR, age, gender, disease status, PMI (post mortem interval), RIN (RNA integrity number), and *APOE* genotype (Table S2). After applying the appropriate covariates to the model, analysis of covariance (ANCOVA) was used to test for association between genotypes and gene expression. SNPs were tested using an additive model. All analyses were performed using statistical analysis software (SAS).

### Replication Dataset

The replication dataset was obtained from Myers et al [15]. Brains were obtained from National Institute on Aging Alzheimer’s Centers and the Miami Brain Bank. The 193 brains came from 18 sites and were composed of 20% frontal lobe, 70% temporal lobe, and 1% parietal lobe. The sample was 46% female with a mean age of 81 (range 65–100) and an average post-mortem interval of 10 hours. Expression levels were measured on an Illumina Human Refseq-8 Expression Bead Chip System. To analyze expression levels, residual values were used that were log transformed and incorporate site, brain region, post-mortem interval, age, *APOE* genotype, and hybridization date as covariates.

## Results

Recent large-scale LOAD GWAS have identified SNPs in *ABCA7*, *BIN1*, *CD2AP*, *CD33*, *CLU*, *CR1*, *EPHA1*, *MS4A6A*, *MS4A4E*, and *PICALM* [5]–[10]. To determine if gene expression is altered in AD, mRNA levels for each gene were measured by real-time PCR in the parietal lobe of AD case and age-matched, cognitively normal, control brains. All gene expression values were normalized to *GAPDH*, a housekeeping gene that accounts for total cell number. Because AD brains are characterized by neuronal loss, reactive gliosis, and microglial activation, we also corrected gene expression levels for specific subpopulations of cells (neurons [*MAP2*], microglia [*AIF1*], and astrocytes [*GFAP*]) to determine if there were cell specific effects on gene expression. *ABCA7* expression was associated with CDR (p = 0.0304), where higher expression levels are correlated with elevated CDR (Table 2). CDR scores increase with cognitive and functional decline [13]. This association remained significant after correcting for subpopulations of cells (Table 2). After correcting expression for neuronal number, *BIN1* expression was associated with age at onset (p = 0.0407) and disease duration (p = 0.0407), where higher expression levels are correlated with later age at onset and shorter disease duration (Table 2). The expression of the neuronal isoform of *BIN1* (*BIN1*n) was also associated with disease duration after correcting for total, neuronal, and microglial cell populations (Table 2). Correcting expression levels for neuronal and microglial cell populations produced significant associations between disease status and CDR with CD33 and CR1 expression (Table 2). Correcting *CLU* expression levels for neuronal number resulted in the association of *CLU* expression with disease status after correcting for neuronal cell populations (p = 0.0159) (Table 2). *CLU* is alternatively spliced into two isoforms [16]. *CLU* isoforms containing exon 5 (*CLU* ^1^) produced similar association patterns after correcting for neuronal and microglia cell populations (Table 2). Additionally, *MS4A6A* expression levels were weakly associated with Braak tangle and Braak plaque scores (p = 0.0564 and p = 0.0559, respectively), where higher expression levels are correlated with higher Braak scores (Table 2). Higher Braak scores are indicative of more extensive tau and amyloid pathology in the brain [11], [12]. The association between *MS4A6A* expression and Braak tangle and Braak plaque scores was slightly stronger after correcting for neuronal expression (p = 0.0437 and 0.0215, respectively; Table 2). Accounting for microglia number revealed an association between *MS4A6A* expression and CDR (p = 0.0311) and Braak tangle score (p = 0.0453). *BIN1, CD2AP, EPHA1*, and *PICALM* expression levels, however, were not associated with AD status or AD pathology (Table 2). Together, we demonstrate that in the absence of strong statistical associations between gene expression and clinical/neuropathological AD outcomes, accounting for subpopulations of cells reveals additional gene expression effects that are likely related to gene function and/or AD-specific cell loss.

**Table 2:** Gene expression is associated with AD pathology.

Gene	Cell Type	Status		Age at Onset		Disease Duration		CDR		Braak Tangle Score		Braak Plaque Score	
		P value	Beta	P value	Beta	P value	Beta	P value	Beta	P value	Beta	P value	Beta
ABCA7	GAPDH	0.2154	0.19	0.5824	0.01	0.5824	−0.01	**0.0304**	0.12	0.2052	0.06	0.3044	0.09
	MAP2	0.1820	0.41	0.4067	−0.01	0.5236	−0.01	**0.0483**	0.12	**0.0247**	0.11	0.0583	0.21
	AIF1	0.1207	0.38	0.7996	0.01	0.7996	−0.01	**0.0260**	0.19	0.2371	0.07	0.7743	0.04
	GFAP	0.8641	0.03	0.7867	−0.01	0.5577	0.02	**0.0324**	0.15	0.2957	0.05	0.9937	0.01
BIN1	GAPDH	0.3490	0.15	0.1006	0.04	0.1006	−0.04	0.3620	−0.05	0.3505	−0.04	0.5084	0.06
	MAP2	0.1146	0.35	**0.0407**	0.06	**0.0407**	−0.06	0.3587	−0.07	0.9638	0.01	0.2273	0.17
	AIF1	0.2104	0.3	0.1638	0.05	0.1638	−0.05	0.9481	−0.01	0.5857	−0.04	0.9815	0.01
	GFAP	0.7398	−0.05	0.4874	0.01	0.7305	−0.01	0.4793	−0.04	0.3235	−0.04	0.9436	−0.01
BIN1n	GAPDH	0.3886	−0.19	0.4042	0.01	**0.0111**	−0.08	0.2006	−0.10	0.4142	0.05	0.1287	0.16
	MAP2	0.8622	0.04	0.2208	0.02	**0.0061**	−0.09	0.2474	−0.1	0.1524	0.09	**0.0194**	0.28
	AIF1	0.9806	−0.01	**0.0368**	0.08	**0.0368**	−0.08	0.7200	−0.04	0.5563	0.04	0.4315	0.11
	GFAP	0.0915	−0.38	0.1047	0.03	0.0861	−0.05	0.2004	−0.11	0.6392	0.03	0.5061	0.08
CD2AP	GAPDH	0.8737	0.02	0.9658	−0.01	0.9658	0.01	0.5370	−0.03	0.2564	−0.04	0.9958	0.01
	MAP2	0.2703	0.2	0.9181	−0.01	0.3334	−0.02	0.4715	−0.05	0.9073	−0.01	0.3219	0.12
	AIF1	0.4028	0.17	0.8332	0.01	0.8332	−0.01	0.7965	0.02	0.4525	−0.04	0.6573	−0.05
	GFAP	0.2235	−0.18	0.5119	−0.01	0.1542	0.03	0.7290	−0.02	0.1924	−0.05	0.4547	−0.07
CD33	GAPDH	0.6291	0.06	0.5261	0.01	0.4950	0.01	0.4612	0.04	0.7889	−0.01	0.7049	0.02
	MAP2	**0.0431**	0.4	0.3260	0.01	0.9951	0.01	0.1730	0.1	0.2093	0.07	0.0753	0.21
	AIF1	**0.0174**	0.27	0.9665	−0.01	0.9665	0.01	**0.0002**	0.15	0.3337	0.03	0.9328	−0.01
	GFAP	0.3917	−0.14	0.0889	−0.04	0.0889	0.04	0.4222	0.05	0.8155	−0.01	0.6516	−0.04
CLU^1^	GAPDH	0.3105	0.09	0.7466	−0.01	0.6191	−0.01	0.3882	0.03	0.8932	−0.01	0.8379	0.01
	MAP2	**0.0159**	0.35	0.5680	0.01	0.2185	−0.02	0.4023	0.05	0.2740	0.04	0.0766	0.16
	AIF1	0.1051	0.27	0.8649	0.01	0.8649	−0.01	0.1269	0.09	0.9266	0.01	0.6945	−0.04
	GFAP	0.4559	−0.09	0.9527	0.01	0.1758	0.02	0.6251	0.02	0.4575	−0.03	0.3505	−0.07
CLU^2^	GAPDH	0.0664	0.15	0.7600	−0.01	0.6676	−0.01	0.1229	0.05	0.3793	0.02	0.5189	0.03
	MAP2	**0.0036**	0.42	0.4485	0.01	0.2147	−0.02	0.2129	0.07	0.1224	0.06	**0.0290**	0.19
	AIF1	**0.0500**	0.33	0.9039	0.01	0.9039	−0.01	0.0689	0.11	0.5898	0.02	0.9498	−0.01
	GFAP	0.8104	−0.03	0.8314	0.01	0.1798	0.03	0.3934	0.04	0.8464	−0.01	0.5848	−0.04
CR1	GAPDH	0.2452	0.25	0.9715	−0.01	0.8680	0.01	0.6467	0.03	0.2183	0.07	0.1598	0.17
	MAP2	**0.0252**	0.59	0.9118	0.01	0.9420	−0.01	0.2813	0.11	0.0551	0.14	0.0553	0.33
	AIF1	**0.0303**	0.51	0.4042	−0.02	0.4042	0.02	**0.0444**	0.17	0.0608	0.11	0.3737	0.13
	GFAP	0.7829	0.05	0.8507	0.01	0.2024	0.04	0.5759	0.04	0.2764	0.05	0.3066	0.11
EPHA1	GAPDH	0.0782	−0.28	0.2299	−0.01	0.9897	0.01	0.2992	−0.06	0.3080	−0.04	0.1947	−0.11
	MAP2	0.8657	0.03	0.4988	−0.01	0.6580	−0.01	0.7552	−0.02	0.6829	0.02	0.8267	0.02
	AIF1	0.9763	0.01	0.8337	−0.01	0.8337	0.01	0.5315	0.05	0.9332	−0.01	0.2446	−0.15
	GFAP	0.0596	0.45	0.8758	−0.01	0.2716	0.03	0.5927	−0.05	0.5900	−0.03	0.1937	−0.18
MS4A6A	GAPDH	0.7251	0.06	0.5184	0.01	0.7308	0.01	0.5719	0.04	0.0564	0.09	0.0559	0.19
	MAP2	0.1844	0.29	0.2252	0.02	0.8651	−0.01	0.5779	0.04	**0.0437**	0.12	**0.0215**	0.29
	AIF1	0.1332	0.25	0.7390	0.01	0.7390	−0.01	**0.0311**	0.13	**0.0453**	0.09	0.1969	0.13
	GFAP	0.4191	−0.15	0.2453	−0.03	0.2453	0.03	0.5131	0.05	0.1440	0.07	0.1891	0.12
PICALM	GAPDH	0.4682	0.10	0.1283	0.03	0.1283	−0.03	0.9328	−0.01	0.7067	−0.01	0.4494	0.06
	MAP2	0.1351	0.29	0.4987	0.01	0.0614	−0.05	0.8770	−0.01	0.5206	0.04	0.1720	0.17
	AIF1	0.2380	0.26	0.2692	0.04	0.2692	−0.04	0.5084	0.05	0.9453	−0.01	0.9737	0.01
	GFAP	0.4669	−0.11	0.9864	0.01	0.9252	−0.01	0.8801	0.01	0.6861	−0.01	0.9317	−0.01
MAP2	GAPDH	**0.0224**	−0.27	0.2440	−0.01	0.3640	0.01	0.5628	−0.03	0.1170	−0.06	0.0773	−0.14
AIF1	GAPDH	0.2354	−0.19	**0.0203**	0.03	0.8856	−0.03	0.1964	−0.07	0.6619	−0.02	0.4672	0.06
GFAP	GAPDH	0.2658	0.19	0.7913	−0.01	0.1785	−0.03	0.9257	−0.01	0.7754	0.01	0.5411	0.06

Covariates included in analyses are reported in Table S2. CLU^1^, probe spans exons 3–4. CLU^2^, probe spans exons 4–5.

**Figure 1:**
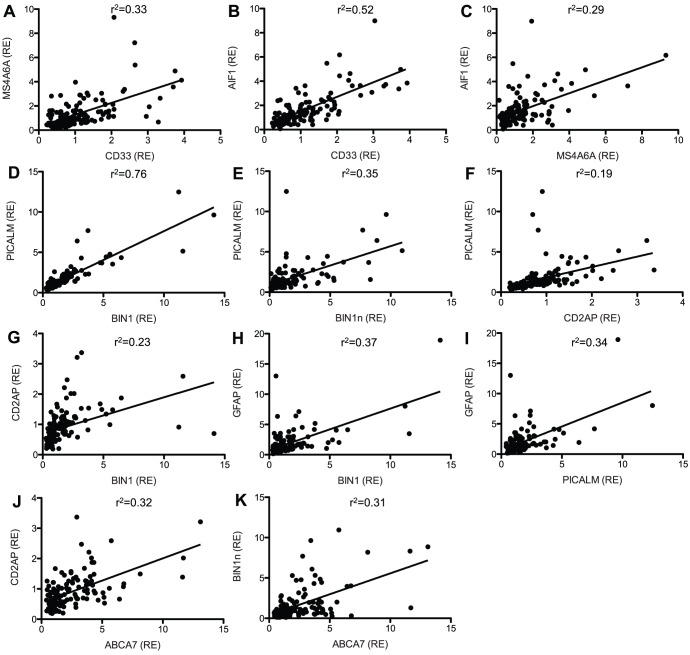
Expression of genes involved in immune response and synaptic function are highly correlated in brain tissue. Relative expression (RE) was plotted for the indicated genes. Genes involved in immune response (A-C). Genes involved in synaptic function (D-I). Genes involved in cholesterol metabolism and synaptic function (J-K).

The top LOAD risk genes fall into three functional categories: immune response (*CLU*, *CR1*, *ABCA7*, *MS4A*, *CD33*, and *EPHA1*), cholesterol metabolism (*CLU* and *ABCA7*), and synaptic function (*PICALM*, *BIN1*, *CD33*, *CD2AP*, and *EPHA1*). We used the expression data for these genes to test whether expression levels of genes in a similar functional class are correlated. Expression of *CD33* and *MS4A6A*, both of which function in immune response, were highly correlated (Figure 1A). Furthermore, expression of *CD33* and *MS4A6A* were highly correlated with *AIF1* expression, a marker for microglia, the immune cell of the brain (Figure 1B–C). Expression of genes related to synaptic function, *BIN1*, *BIN1*n, *CD2AP*, and *PICALM*, were highly correlated (Figure 1D–G). *BIN1* and *PICALM* expression were also highly correlated with *GFAP* expression, an astrocytic marker (Figure 1H–I). *ABCA7* expression, involved in immune response and cholesterol metabolism, was highly correlated with *BIN1* and *CD2AP* expression, which are involved in synaptic function (Figure 1J–K). Together, these results demonstrate that genes that fall into the same functional category are related at the RNA level. Thus, their dysfunction may be linked in AD.

To determine if the LOAD GWAS SNPs influence gene expression, we analyzed the association of SNP genotype with gene expression using an ANCOVA and testing for association with an additive model, the model utilized when originally reporting association between these SNPs and risk for AD [5]–[10]. We failed to detect an association between GWAS SNPs and cis-acting expression quantitative trait loci (eQTL) after correcting for the total cell population (Table 3) or specific cell types (Table S3).

**Table 3:** AD GWAS SNPs do not modify gene expression in the parietal lobe of human brains.

SNP	Gene	MAF	P value	Beta
rs3764650	ABCA7	0.13	0.6471	0.07
rs744373	BIN1	0.34	0.7720	0.03
rs59335482	BIN1	0.31	0.2879	0.12
rs744373	BIN1n	0.34	0.2666	0.17
rs59335482	BIN1n	0.31	0.1217	0.24
rs9349407	CD2AP	0.26	0.0610	−0.18
rs3865444	CD33	0.29	0.3071	0.10
rs7982	CLU^1^	0.38	0.1324	−0.09
rs7982	CLU^2^	0.38	0.1452	−0.08
rs670173	CR1	0.01	0.9630	−0.02
rs3818361	CR1	0.22	0.1753	−0.20
rs11767557	EPHA1	0.16	0.1989	0.17
rs610932	MS4A6A	0.43	0.5130	−0.13
rs3851179	PICALM	0.38	0.2791	−0.09

Covariates included in analyses are reported in Table S2.

**Figure 2:**
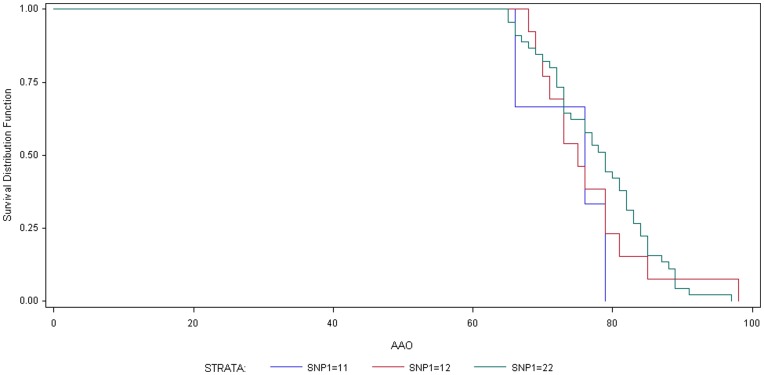
Rs3764650 in *ABCA7* is associated with age at onset. Kaplan-Meier curve. AAO, age at onset in years. SNPs were analyzed using an additive model. G, minor allele. Blue line, TT (11). Red line, TG (12). Green line, GG (22).

LOAD GWAS SNPs were identified based on their association with disease status. To determine if these SNPs contribute to AD pathology, independent of gene expression, we analyzed the association of each SNP with clinical (disease status, age at onset, disease duration, and CDR) and neuropathological (Braak tangle and Braak plaque score) measures of AD. The minor allele of rs3764650 in *ABCA7* was associated with a later age at onset and shorter disease course (p = 0.0040, p = 0.0040, respectively; Table 4; Figure 2). The minor allele of rs670139 in *MS4A6E* was associated with Braak tangle and Braak plaque score (p = 0.0411, p = 0.0581, respectively; Table 4). We failed to detect an association between the remaining GWAS SNPs and the clinical/neuropathological measures of AD (Table 4).

**Table 4:** Gene SNPs do not significantly influence AD brain pathology.

		Status*		CDR°§		Age at Onset*∧		Disease Duration*∧		Braak Tangle Score*∧		Braak Plaque Score*∧	
SNP	Gene	P value	Beta	P value	Beta	P value	Beta	P value	Beta	P value	Beta	P value	Beta
rs3764650	ABCA7	0.1524	−0.13	0.2966	1.87	**0.0040**	2.58	**0.0040**	−2.58	0.5875	−0.18	0.7356	−0.11
rs744373	BIN1	0.8570	−0.01	0.2245	1.85	0.5234	0.49	0.5234	−0.49	0.4395	−0.21	0.4194	0.21
rs59335482	BIN1	0.7857	0.02	0.3509	1.38	0.9192	0.08	0.9192	−0.08	0.1477	−0.38	0.5852	0.14
rs9349407	CD2AP	0.8372	0.02	0.1217	−2.52	0.0507	−1.56	0.0507	1.56	0.5909	0.16	0.4127	0.20
rs3865444	CD33	0.4485	0.06	0.8427	−0.34	0.4515	−0.64	0.4515	0.64	0.6188	−0.15	0.9381	0.02
rs7982	CLU	0.8533	−0.01	0.9251	−0.14	0.4431	0.57	0.4431	−0.57	0.8560	0.05	0.1357	−0.34
rs670173	CR1	0.3697	−0.18	0.3205	3.62	0.7683	−0.52	0.7683	0.52	N/A	N/A	N/A	N/A
rs3818361	CR1	0.3315	−0.08	0.9699	−0.07	0.2829	−0.95	0.2829	0.95	0.2082	−0.35	0.6167	0.13
rs11767557	EPHA1	0.3221	−0.08	0.4902	−1.19	0.7123	0.31	0.7123	−0.32	0.1470	−0.45	0.8202	0.07
rs610932	MS4A6A	0.2860	0.07	0.9290	−0.12	0.1532	0.97	0.1532	−0.97	0.1520	0.34	0.0998	0.43
rs670139	MS4A6E	0.1624	−0.09	0.5966	0.82	0.9753	−0.02	0.9753	0.02	**0.0411**	−0.53	0.0581	−0.47
rs3851179	PICALM	0.6162	0.03	0.6355	−0.73	0.3357	−0.74	0.3357	0.74	0.8133	0.06	0.1133	0.41

Covariates included in the analysis are marked: *Age, °APOE, §PMI, ∧CDR.

To replicate our findings, we analyzed a publically available AD dataset [15], in which RNA was measured by the Illumina Human Refseq-8 Expression Bead Chip System. Of the nine genes analyzed in our cohort, only five survived quality control measures in the replication dataset: *ABCA7, BIN1, CLU, MS4A6A*, and *PICALM*. We analyzed residual expression levels for association with disease status. *MS4A6A* and *CLU* expression levels were significantly associated with disease status (p = 0.0346 and p = 0.0334, respectively), where *MS4A6A* and *CLU* expression was up regulated in the AD brains compared with controls (Table 5). *BIN1* expression levels were marginally associated with disease status (p = 0.0540), where expression was also up regulated in AD brains compared with controls (Table 5).

**Table 5:** CLU and MS4A6A expression are associated with AD status in a replication dataset.

	Status	
Gene	P value	Beta
ABCA7	0.3471	0.07
BIN1	0.0540	0.09
CLU	**0.0334**	0.11
MS4A6A	**0.0346**	0.19
PICALM	0.1405	0.09

## Discussion

AD is the most common form of dementia. AD etiology is influenced by complex interactions between genetic and environmental risk factors. *APOE4* is the strongest risk factor for LOAD; however, variation in *APOE* accounts for only 10–20% of LOAD risk, suggesting that additional risk genes exist for LOAD. Recent LOAD GWAS genes have been identified that are involved in cholesterol metabolism, synaptic function, and immune response. Yet, the functional impact of these genes in LOAD remains to be determined. In this study, we measured the influence of LOAD GWAS SNPs and gene expression levels on clinical and neuropathological measures of AD in parietal brain tissue from AD cases and cognitively normal individuals. *ABCA7, BIN1, CD33, CLU, and CR1* expression levels were associated with clinical measures of AD (disease status, age at onset, disease duration, and/or CDR), and *MS4A6A* expression levels were associated with neuropathological measures of AD (Braak tangle and Braak plaque score). We failed to detect an association between GWAS SNPs and gene expression levels. We found that the minor allele of rs3764650 in *ABCA7* was associated with clinical measures of AD (age at onset and disease duration), and the minor allele of rs670139 in *MS4A6E* is associated with neuropathological (Braak tangle and Braak plaque score) measures of AD. Together, these findings demonstrate that *ABCA7, BIN1, CD33, CLU, CR1,* and the *MS4A* gene family are affected at the mRNA level in AD brains.

### *ABCA7*, *BIN1*, *CD33*, *CLU* Gene Family Expression are Marginally Associated with AD Phenotypes, *CR1*, and *MS4A*

In this study, we found that *ABCA7* expression levels are significantly associated with CDR, with higher expression levels of this gene being correlated with more extensive cognitive decline. We also demonstrated that the minor allele of rs3764650 in *ABCA7* was associated with age at onset and disease duration, where the minor allele was associated with later age at onset and shorter disease duration. ABCA7 is an ATP-binding cassette transporter protein [17]–[19]. ABCA7 transports xenobiotics, metals, inorganic ions, carbohydrates, vitamins, amino acids, peptides, and lipids [20]–[23]. ABCA7 is highly expressed in the CA region of the hippocampus [24], where microglia express the protein at levels ten times greater than is observed in neurons [25]. ABCA7 has been predicted to stimulate the cellular cholesterol efflux to a lipid-free acceptor. ABCA7 may also play a role in phagocytosis [26].

*BIN1* and the neuron specific *BIN1* isoform (*BIN1*n) expression levels were associated with clinical measures of AD, where elevated expression was associated with later age at onset and shorter disease duration. Bin1 is implicated in receptor-mediated endocytosis and recycling of endosomes in the cell. Bin1 knockout mice do not exhibit deficiency in synaptic vesicle recycling [27], [28] but have less age-associated inflammation [29].

*CD33* and *CR1* expression levels were associated with clinical measures of AD, where elevated expression levels were associated with AD after correcting for neuron and microglia number in the brain. CD33 and CR1 function in immune response pathways. CD33 is a transmembrane receptor expressed on cells from the myeloid lineage. CD33 functions in the innate and adaptive immune response [30], and it may play a role in receptor-mediated endocytosis independent of clathrin [31]. CR1 plays an essential role in the adaptive immune response. CR1 is highly expressed in red blood cells [32], where it mediates cell binding to particles and immune complexes. CR1 is a negative regulator of the complement cascade; mediates immune adherence and phagocytosis; and inhibits the classical and alternative complement pathways [33].

*CLU* expression levels are associated with clinical measures of AD, where elevated *CLU* levels occur in individuals with AD. Clusterin (ApoJ) exists as two isoforms and is highly expressed in astrocytes [16]. Clusterin is secreted from cells where it is reported to have several roles in the cell: chaperone function [34], [35], lipid trafficking [36], [37], and inhibition of the complement cascade [38]. Clusterin inhibits complement activation and the membrane attack complex [38], which is relevant to AD in that neuroinflammation is a key feature of the disease. Clusterin has been implicated in AD in its ability to assist in refolding of misfolded proteins [35], bind to fibrillar proteins [39], [40], clearance of Aβ [41], and interact with ApoE [41]. Neuritic dystrophy and fibrillar amyloid deposits are markedly reduced when *CLU* is knocked out in PDAPP mice [42], suggesting that *CLU* may have deleterious effects when upregulated in AD brains. However, in the absence of APOE and CLU, PDAPP mice have accelerated disease onset, elevated CSF and ISF beta-amyloid levels, and more extensive amyloid deposition in the brain [41]. Thus, the role of clusterin in the brain is complex and influenced by other genes.

In our cohort, genes in the *MS4A* gene cluster showed association with clinical and neuropathological measures of AD. *MS4A6A* expression levels were found to be associated with elevated Braak tangle and Braak plaque scores. Additionally, the minor allele of rs670139 in *MS4A6E* was associated with CDR, Braak tangle score, and Braak plaque score. The MS4A family of genes is reported to play a role in the immune response via expression on high affinity IgE receptors [43]; however, little is known about the function of each family member. While several genes in the *MS4A* gene cluster have been identified in recent LOAD GWAS [9], [10], we only measured expression levels of the *MS4A6A* gene. Due to extensive sequence conservation between the *MS4A* genes, we were unable to identify Taqman probes in other *MS4A* genes that would specifically detect a single gene; thus, we are limited in our interpretation of the role of each of the *MS4A* genes in AD brains. While our replication data set only contained the *MS4A6A* gene, we were able to replicate the association with disease status.

### Factors Contributing to the Absence of Robust Findings

The associations we describe in this study are only marginal and would not survive multiple test correction. We interpret these findings to point to subtle effects in gene expression. However, type 1 errors are also a possible explanation. Our observations that the association of gene expression with clinical and neuropathological measures of AD can change after correction for neuronal, astrocytic, and microglial subpopulations indicates that cell specific gene expression plays an important role in disease.

We chose to examine measures of AD (disease status, CDR, Braak plaque score and Braak tangle score) because each trait represents a different, not completely overlapping, aspect of Alzheimer’s disease. AD status, a dichotomous trait, is assigned at autopsy based on several criteria, including clinical dementia, neuronal death and Braak plaque and Braak tangle scores. CDR, however, represents a clinical diagnosis that measures six domains of cognitive and functional abilities including memory, orientation, problem solving, community involvement, home and personal care. The CDR trait differs from AD status in that it is an ordinal trait that describes disease severity. Similarly, Braak plaque and tangle scores are also ordinal traits, each representing an aspect of AD pathology. Because disease status is a dichotomous trait, while the other phenotypes are ordinal, it remains possible that the absence of association across phenotypes is an issue of statistical power.

We failed to detect expression differences in the clinical and neuropathological measurements of AD in some of the genes tested in this study. These findings do not eliminate the possibility that changes are occurring in these genes during disease that we are unable to capture in our cohort. With a sample size of 112, this study may be underpowered to observe more subtle changes in gene expression that could contribute to LOAD. Furthermore, our study was limited to the parietal lobe, where AD pathology occurs late in the disease. It is possible that testing other brain regions that are susceptible to AD pathology at earlier time points in the disease course could produce additional associations. Environmental factors may also contribute to or obscure gene expression levels; however, at this time, we do not possess adequate phenotypic data to analyze this properly.

### The Complexities of Defining the Functional Impact of LOAD GWAS SNPs

In this study, we analyzed genotype association with gene expression level to determine if the LOAD GWAS SNPs were functionally relevant. We failed to identify any SNPs that influence gene expression levels independent of disease status. Thus, it is possible that the functional polymorphisms that exist within these genes are rare, alter gene splicing, or impact inducible expression rather than constitutive expression. These findings fit with our previous study: we were unable to identify statistically significant associations of GWAS SNPs and SNPs in linkage disequilibrium with GWAS SNPs with CSF tau and Aβ levels [44]. Thus, it is essential to exploit deep sequencing techniques to identify functional variants in these genes.

### LOAD GWAS Genes are Functionally Linked

The genes identified in recent LOAD GWAS fall into three functional categories: immune response (*CLU*, *CR1*, *ABCA7*, *MS4A* family, *CD33*, and *EPHA1*), cholesterol metabolism (*CLU* and *ABCA7*), and synaptic function (*PICALM*, *BIN1*, *CD33*, *CD2AP*, and *EPHA1*). The genes with the most significant association with clinical and neuropathological measures of AD function in immune response and cholesterol metabolism. Despite an absence of association with the remaining GWAS genes, it is possible that these genes are affected at the protein level in AD brains.

Changes in genes that influence immune response may be difficult to identify in autopsied brain tissue, as the immune response can be transient. Additionally, alterations of the immune response in AD may primarily occur in organs other than the brain. CD2AP is localized in the cytoplasm where it has several functions: cytoskeletal remodeling [45]; cell survival [46], [47]; endocytosis [48]–[50]. CD2AP functions in the immune response by interacting with CD2, a T-cell and natural killer cell membrane protein, and facilitates T-cell adhesion to antigen-presenting cells [45].

The influence of GWAS SNPs and their corresponding genes in AD that are associated with synaptic function may be more apparent at the protein level. Picalm functions in receptor-mediated endocytosis where it is essential in clathrin assembly, axogenesis, and dendritic outgrowth in neurons [51]. EphA1 is highly expressed in the adult brain, where it participates in forward signaling in receptor-bearing cells and reverse signaling in ligand-bearing cells by binding to GPI-linked A ephrins, which together facilitates axon guidance and communication between neighboring cell populations [52]–[57]. CD2AP knockout also mice exhibit deficiencies in receptor trafficking to the lysosome.

### Conclusions

This study provides evidence for the involvement of *ABCA7*, *BIN1*, *CD33*, *CLU*, *CR1*, and *MS4A* gene family in AD brain pathology. As AD is a complex disorder, it is likely that many genes are affected at the RNA and protein levels and that an understanding of the complex interactions that may occur between these genes is essential to understanding and treating AD.

## Supporting Information

Figure S1 **C_T_ values for expression assays.** Non-normalized C_T_ values for each gene expression assay were averaged for AD (white) and non-demented control (black) brains.
(PDF)

Figure S2 ***GAPDH*** **and** ***PPIA*** **expression are highly correlated.** Average C_T_ was plotted for each sample. A. GAPDH-FAM versus PPIA. B. GAPDH-VIC versus PPIA C. GAPDH-FAM versus GAPDH-VIC.
(PDF)

Figure S3 **Normalization of gene expression by log transformation.** Log transformed values of relative expression values for each LOAD GWAS genes are illustrated in a histogram. Red line, normal density curve. Gray line, fitted density curve.
(PDF)

Table S1 **Average RNA integrity number for case and control brains.**
(DOCX)

Table S2 **Covariates that were included in analysis.**
(DOCX)

Table S3 **AD GWAS SNPs do not modify gene expression in the parietal lobe of human brains after correcting for cell-specific gene expression.**
(DOCX)
