# Supplementary figures and images for: Expression of Novel Alzheimer’s Disease Risk Genes in Control and Alzheimer’s Disease Brains
Source: PLoS One. 2012 Nov 30;7(11):e50976. doi: 10.1371/journal.pone.0050976 (PMC3511432; doi:10.1371/journal.pone.0050976)

Supplemental Figure 1

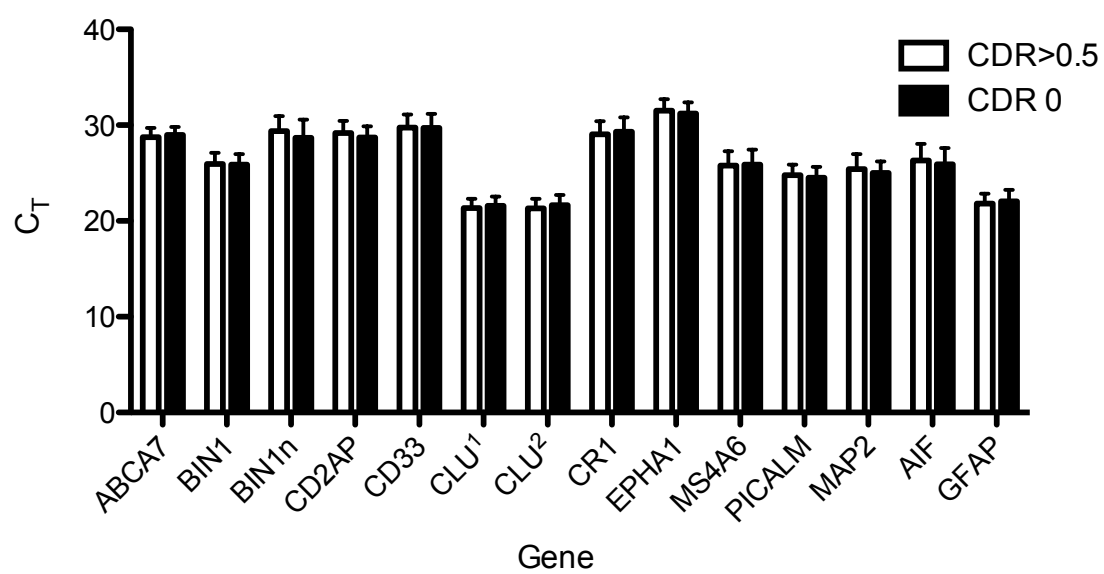

Supplement: Figure S1 — CT values for expression assays. Non-normalized CT values for each gene expression assay were averaged for AD (white) and non-demented control (black) brains. (PDF) [file pone.0050976.s001.pdf]

## Supplemental Figure 2

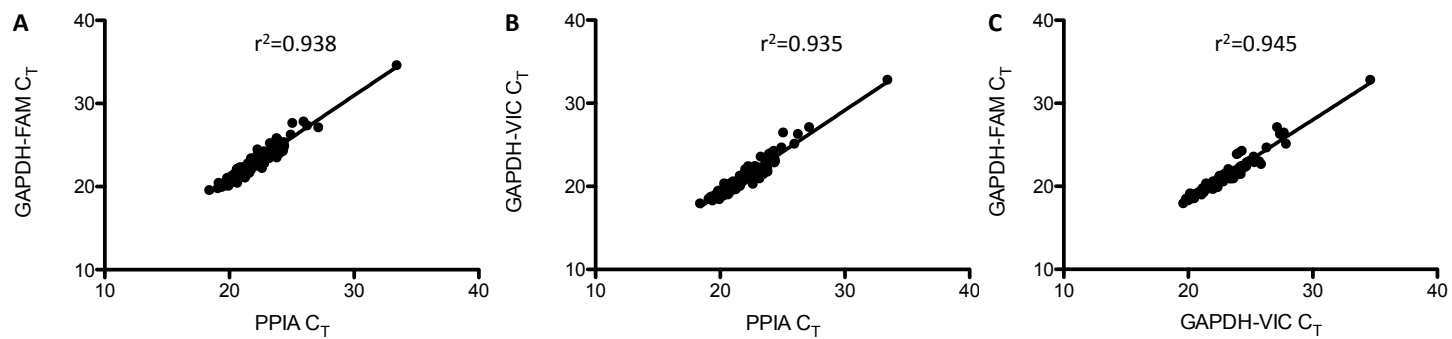

Supplement: Figure S2 — GAPDH and PPIA expression are highly correlated. Average CT was plotted for each sample. A. GAPDH-FAM versus PPIA. B. GAPDH-VIC versus PPIA C. GAPDH-FAM versus GAPDH-VIC. (PDF) [file pone.0050976.s002.pdf]

# Supplemental Figure 3

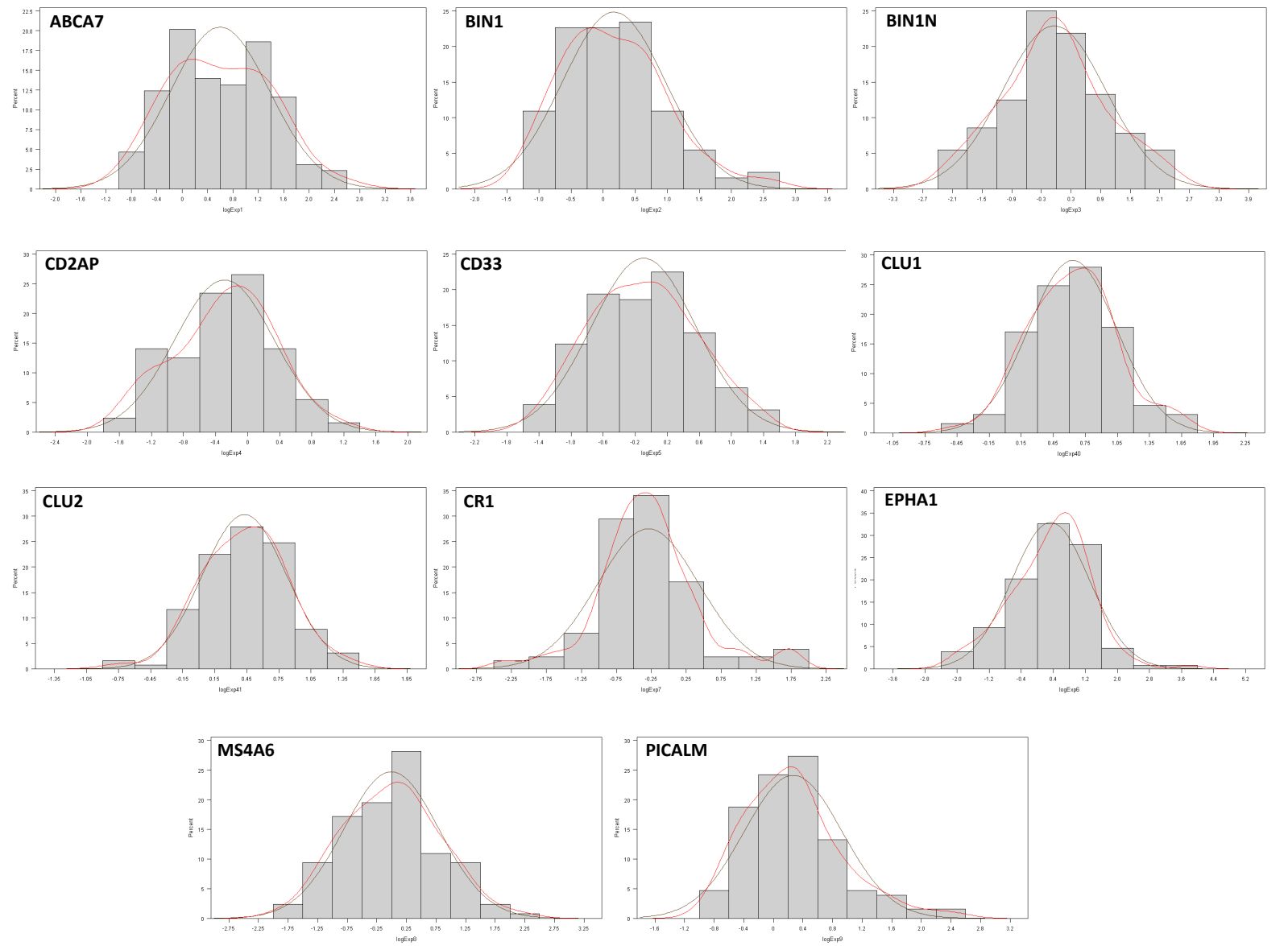

Supplement: Figure S3 — Normalization of gene expression by log transformation. Log transformed values of relative expression values for each LOAD GWAS genes are illustrated in a histogram. Red line, normal density curve. Gray line, fitted density curve. (PDF) [file pone.0050976.s003.pdf]
