# Supplementary material for: Expression of Novel Alzheimer’s Disease Risk Genes in Control and Alzheimer’s Disease Brains
Source: PLoS One. 2012 Nov 30;7(11):e50976. doi: 10.1371/journal.pone.0050976 (PMC3511432; doi:10.1371/journal.pone.0050976)
Supplement: Table S1 — Average RNA integrity number for case and control brains. (DOCX) [file pone.0050976.s004.docx]

Table S1: Average RNA integrity number for case and control brains

| Disease Status | RIN Mean (±STD) |
| --- | --- |
| Case | 5.2±1.4 |
| Control | 5.2±1.5 |
