# Supplementary material for: Expression of Novel Alzheimer’s Disease Risk Genes in Control and Alzheimer’s Disease Brains
Source: PLoS One. 2012 Nov 30;7(11):e50976. doi: 10.1371/journal.pone.0050976 (PMC3511432; doi:10.1371/journal.pone.0050976)
Supplement: Table S2 — Covariates that were included in analysis. (DOCX) [file pone.0050976.s005.docx]

Table S2: Covariates that were included in analysis.

| Gene | Gene Correction | Covariates |
| --- | --- | --- |
| ABCA7 | GAPDH | Age, PMI, CDR |
| BIN1 | GAPDH | Age |
| BIN1n | GAPDH | PMI |
| CD2AP | GAPDH | Age |
| CD33 | GAPDH | None |
| CLU^1^ | GAPDH | None |
| CLU^2^ | GAPDH | Status |
| CR1 | GAPDH | None |
| EPHA1 | GAPDH | ApoE, PMI, CDR |
| MS4A6 | GAPDH | None |
| PICALM | GAPDH | Age |
| MAP2 | GAPDH | ApoE PMI, CDR |
| AIF1 | GAPDH | Age, PMI |
| GFAP | GAPDH | None |
| ABCA7 | MAP2 | PMI, status |
| BIN1 | MAP2 | PMI, CDR, age |
| BIN1-N | MAP2 | PMI |
| CD2AP | MAP2 | PMI |
| CD33 | MAP2 | Status, gender, ApoE |
| CLU^1^ | MAP2 | Status, ApoE, PMI |
| CLU^2^ | MAP2 | Status, ApoE, PMI |
| CR1 | MAP2 | Status, ApoE |
| EPHA1 | MAP2 | PMI |
| MS4A6 | MAP2 | None |
| PICALM | MAP2 | PMI, CDR |
| ABCA7 | AIF1 | Age, CDR, PMI |
| BIN1 | AIF1 | Age, PMI |
| BIN1-N | AIF1 | Age, PMI |
| CD2AP | AIF1 | Age, PMI |
| CD33 | AIF1 | CDR, age, gender |
| CLU^1^ | AIF1 | Age, CDR, PMI |
| CLU^2^ | AIF1 | Age, CDR, PMI |
| CR1 | AIF1 | Age, status, ApoE |
| EPHA1 | AIF1 | Age, PMI |
| MS4A6 | AIF1 | CDR, age |
| PICALM | AIF1 | Age, PMI |
| ABCA7 | GFAP | CDR, gender |
| BIN1 | GFAP | None |
| BIN1-N | GFAP | Gender, status, PMI |
| CD2AP | GFAP | None |
| CD33 | GFAP | Age |
| CLU^1^ | GFAP | Gender |
| CLU^2^ | GFAP | Gender |
| CR1 | GFAP | None |
| EPHA1 | GFAP | ApoE, status, gender |
| MS4A6 | GFAP | Age, gender |
| PICALM | GFAP | None |
