# Supplementary material for: Expression of Novel Alzheimer’s Disease Risk Genes in Control and Alzheimer’s Disease Brains
Source: PLoS One. 2012 Nov 30;7(11):e50976. doi: 10.1371/journal.pone.0050976 (PMC3511432; doi:10.1371/journal.pone.0050976)
Supplement: Table S3 — AD GWAS SNPs do not modify gene expression in the parietal lobe of human brains after correcting for cell-specific gene expression. (DOCX) [file pone.0050976.s006.docx]

Table S3: AD GWAS SNPs do not modify gene expression in the parietal lobe of human brains after correcting for cell-specific gene expression.

| Expression | Gene Correction | SNP | P value | Beta |
| --- | --- | --- | --- | --- |
| ABCA7 | MAP2 | rs3764650 | 0.2668 | 0.18 |
| BIN1 | MAP2 | rs744373 | 0.9029 | 0.02 |
| BIN1 | MAP2 | rs59335482 | 0.3432 | 0.14 |
| BIN1n | MAP2 | rs744373 | 0.4249 | 0.13 |
| BIN1n | MAP2 | rs59335482 | 0.1944 | 0.22 |
| CD2AP | MAP2 | rs9349407 | 0.5273 | -0.08 |
| CD33 | MAP2 | rs3865444 | 0.9438 | -0.01 |
| CLU^1^ | MAP2 | rs7982 | 0.5544 | -0.06 |
| CLU^2^ | MAP2 | rs7982 | 0.6716 | -0.04 |
| CR1 | MAP2 | rs670173 | 0.9653 | -0.02 |
| CR1 | MAP2 | rs3818361 | 0.3236 | -0.2 |
| EPHA1 | MAP2 | rs11767557 | 0.4840 | 0.09 |
| MS4A6 | MAP2 | rs610932 | 0.7770 | -0.03 |
| MS4A6 | MAP2 | rs670139 | 0.7374 | -0.05 |
| MS4A6 | MAP2 | rs1051756 | 0.2932 | -0.15 |
| PICALM | MAP2 | rs3851179 | 0.4960 | -0.09 |
| ABCA7 | AIF1 | rs3764650 | 0.7607 | -0.07 |
| BIN1 | AIF1 | rs744373 | 0.8854 | -0.03 |
| BIN1 | AIF1 | rs59335482 | 0.7793 | 0.05 |
| BIN1n | AIF1 | rs744373 | 0.8183 | 0.05 |
| BIN1n | AIF1 | rs59335482 | 0.5016 | 0.13 |
| CD2AP | AIF1 | rs9349407 | 0.5767 | -0.08 |
| CD33 | AIF1 | rs3865444 | 0.1104 | 0.13 |
| CLU^1^ | AIF1 | rs7982 | 0.0925 | -0.19 |
| CLU^2^ | AIF1 | rs7982 | 0.1142 | -0.18 |
| CR1 | AIF1 | rs670173 | 0.5697 | -0.27 |
| CR1 | AIF1 | rs3818361 | 0.4394 | -0.14 |
| EPHA1 | AIF1 | rs11767557 | 0.9742 | -0.01 |
| MS4A6 | AIF1 | rs610932 | 0.0677 | -0.19 |
| MS4A6 | AIF1 | rs670139 | 0.9437 | -0.01 |
| MS4A6 | AIF1 | rs1051756 | 0.1087 | -0.19 |
| PICALM | AIF1 | rs3851179 | 0.3868 | -0.13 |
| ABCA7 | GFAP | rs3764650 | 0.9602 | 0.01 |
| BIN1 | GFAP | rs744373 | 0.1567 | -0.15 |
| BIN1 | GFAP | rs59335482 | 0.2259 | -0.13 |
| BIN1n | GFAP | rs744373 | 0.9421 | -0.01 |
| BIN1n | GFAP | rs59335482 | 0.7689 | 0.05 |
| CD2AP | GFAP | rs9349407 | 0.2164 | -0.14 |
| CD33 | GFAP | rs3865444 | 0.5920 | -0.07 |
| CLU^1^ | GFAP | rs7982 | 0.5414 | -0.05 |
| CLU^2^ | GFAP | rs7982 | 0.6574 | -0.04 |
| CR1 | GFAP | rs670173 | 0.6276 | 0.22 |
| CR1 | GFAP | rs3818361 | 0.0862 | -0.27 |
| EPHA1 | GFAP | rs11767557 | 0.8929 | -0.03 |
| MS4A6 | GFAP | rs610932 | 0.4206 | -0.09 |
| MS4A6 | GFAP | rs670139 | 0.9441 | -0.01 |
| MS4A6 | GFAP | rs1051756 | 0.4510 | -0.11 |
| PICALM | GFAP | rs3851179 | 0.4591 | -0.07 |

Association was measured using an additive model. Covariates included in the model are reported in Table S2.
